# 不同基因分型晚期非小细胞肺癌患者的预后分析

**DOI:** 10.3779/j.issn.1009-3419.2017.11.04

**Published:** 2017-11-20

**Authors:** 平 刘, 羽华 吴, 立娟 周, 娜 秦, 权 张, 卉 张, 曦 李, 新勇 张, 嘉林 吕, 新杰 杨, 敬慧 王, 树才 张

**Affiliations:** 1 101149 北京，首都医科大学附属北京胸科医院，北京市结核病胸部肿瘤研究所肿瘤内科 Department of Medical Oncology, Beijing Chest Hospital, Capital Medical University, Beijing Tuberculosis and Thoracic Tumor Research Institute, Beijing 101149, China; 2 101149 北京，首都医科大学附属北京胸科医院，北京市结核病胸部肿瘤研究所病理科 Department of Pathology, Beijing Chest Hospital, Capital Medical University, Beijing Tuberculosis and Thoracic Tumor Research Institute, Beijing 101149, China

**Keywords:** 肺肿瘤, 表皮生长因子受体, 间变性淋巴瘤激酶, 预后, Lung neoplasms, Epidermal growth factor receptor, Anaplastic lymphoma kinase, Prognosis

## Abstract

**背景与目的:**

非小细胞肺癌（non-small cell lung cancer, NSCLC）已由原来的组织分型指导下的治疗转变为基因分型指导治疗的模式，表皮生长因子受体（epidermal growth factor receptor, *EGFR*）和间变性淋巴瘤激酶（anaplastic lymphoma kinase, *ALK*）是肺癌最重要的两个驱动基因。本研究旨在探讨不同基因分型的复发或转移晚期NSCLC患者的临床特点及预后影响因素。

**方法:**

回顾性分析北京胸科医院2004年7月-2015年12月间553例*EGFR*和*ALK*基因状态明确的晚期NSCLC患者的临床资料，采用*Cox*比例风险回归模型对患者预后的独立影响因素进行分析。

**结果:**

553例细胞学或组织学证实的晚期NSCLC患者，*EGFR*突变患者227例，ALK阳性患者58例，*EGFR*和*ALK*双突变患者2例，EGFR和ALK野生型患者266例。227例*EGFR*突变患者的中位生存期（overall survival, OS）为28.7个月（95%CI: 22.160-35.240），体能状态（performance status, PS）评分为0分-1分（HR=4.451; 95%CI: 2.112-9.382; *P* < 0.001）、接受EGFR-酪氨酸激酶抑制剂（tyrosine kinase inhibitors, TKIs）靶向治疗（HR=2.785; 95%CI: 1.871-4.145; *P* < 0.001）是*EGFR*突变患者生存的独立影响因素。58例ALK阳性患者的中位OS为15.5个月（95%CI: 10.991-20.009），接受克唑替尼靶向治疗（*P*=0.022）是ALK阳性患者生存的独立影响因素。266例野生型患者的中位OS为12.1个月（95%CI: 10.660-13.540），PS评分为0分-1分（HR=2.313; 95%CI: 1.380-3.877; *P*=0.001）、接受化疗（HR=1.911; 95%CI: 1.396-2.616; *P* < 0.001）是野生型患者生存的独立影响因素。

**结论:**

不同基因型的晚期NSCLC患者的预后差异较大，靶向治疗可改善*EGFR*突变、ALK阳性患者生存。

## Abstract (en)

**Background and objective:**

Non-small cell lung cancer (NSCLC) has been transformed from the treatment according to histological type to genotype treatment model. The epidermal growth factor receptor (*EGFR*) and anaplastic lymphoma kinase (*ALK*) genes are the most important drivers in lung cancer. The aim of this study is to explore the clinical characteristics and prognostic factors of patients with advanced NSCLC with different genotypes.

**Methods:**

We retrospectively reviewed the clinical data of 553 advanced NSCLC patients with *EGFR* mutations and *ALK* positive who were hospitalized in the Beijing Chest Hospital from July 2004 to December 2015, and the independent prognostic factors of patients were analyzed by *Cox* proportional hazards regression model.

**Results:**

The clinical data of 553 patients (227 with *EGFR* mutations, 58 with ALK positive, 2 with *EGFR* and *ALK* co-mutation and 266 with wild-type) with advanced NSCLC were enrolled in this study. The median survival time of 227 patients with *EGFR* mutations was 28.7 mo (95%CI: 22.160-35.240), and the performance status (PS) score (0-1) (HR=4.451; 95%CI: 2.112-9.382; *P* < 0.001) and EGFR-tyrosine kinase inhibitors (TKIs) targeted therapy (HR=2.785; 95%CI: 1.871-4.145; *P* < 0.001) were the independent prognostic factors for the survival of patients harboring EGFR mutations. The median survival time of 58 patients with ALK positive was 15.5 mo (95%CI: 10.991-20.009), and treatment with crizotinib (*P*=0.022) was the independent influence factor for the survival of ALK positive patients. The median survival time of 266 patients with wild-type was 12.1 mo (95%CI: 10.660-13.540), and the PS score (0-1) (HR=2.313; 95%CI: 1.380-3.877; *P*=0.001) and treatment with chemotherapy (HR=1.911; 95%CI: 1.396-2.616; *P* < 0.001) were the independent prognostic factors for the survival of wild-type patients.

**Conclusion:**

The prognosis of patients with advanced NSCLC is associated with genetic mutation, and targeted therapy has a improvement on survival for patients with *EGFR* mutations or *ALK* rearrangement.

肺癌是当前世界上最常见的恶性肿瘤之一，发病率呈逐年上升趋势，发病率与死亡率居于世界恶性肿瘤之首^[1]^，每年有超过120万人死于肺癌^[2]^。尽管目前肺癌的治疗方法较前有了很大的发展，但5年生存率仍不容乐观，发达国家不足20%，发展中国家情况更为严峻^[3]^，其中一个重要的原因是肺癌患者在确诊时80%已经处于疾病晚期。近十年来，肿瘤分子遗传学发展迅速，尤其是驱动基因的研究取得重大进展，表皮生长因子受体（epidermal growth factor receptor, *EGFR*）和间变性淋巴瘤激酶（anaplastic lymphoma kinase, *ALK*）是肺癌最重要的两个驱动基因。针对驱动基因的靶向药物的研发，改变了晚期非小细胞肺癌（non-small cell lung cancer, NSCLC）的治疗模式，对患者的治疗和预后方面产生了引人注目的影响。针对*EGFR*突变的NSCLC患者，IPASS、WJTOG3405、OPTIMAL、EURTAC、LUX-Lung6等^[4-8]^临床试验研究数据表明EGFR酪氨酸激酶抑制剂（tyrosine kinase inhibitors, TKIs）能显著延长EGFR敏感突变晚期NSCLC患者的客观缓解率（objective response rate, ORR）和无进展生存期（progression-free survival, PFS），并且与传统化疗药物相比，患者的耐受性更好；针对ALK阳性NSCLC患者，克唑替尼是其特异性的TKIs，PROFILE1001、PROFILE1005、PROFILE1007和PROFILE1014^[9-12]^临床试验结果表明克唑替尼能显著提高ALK阳性晚期NSCLC患者的ORR，并且能显著延长患者的PFS。

本研究回顾性分析我院553例EGFR和ALK状态明确的晚期NSCLC患者的临床资料，对不同基因型患者的临床特征、生存时间以及影响患者预后因素进行分析。

## 1 资料与方法

### 1.1 临床资料

回顾性分析我院2004年7月-2015年12月收治的晚期NSCLC患者，病例纳入标准：男女不限；患者为术后复发或晚期肺癌；组织学或细胞学检查证实为NSCLC；组织或细胞块进行EGFR、ALK检测；有详细的临床治疗信息，治疗资料齐全，确实可靠。排除标准：术后未复发患者；组织学或细胞学检查证实为小细胞肺癌；合并其他肿瘤患者；失访患者。癌症分期根据美国癌症联合会（American Joint Commission for Cancer, AJCC）第七版的肺癌肿瘤-淋巴结-转移（tumor-node-metastasis, TNM）分期标准进行分期^[13]^。患者的临床资料包括年龄、性别、吸烟史、体能状态（performance status, PS）评分、病理类型、分期、基因分型及治疗情况等。基因分型方法：*EGFR*突变基因由突变扩增阻滞系统（amplification refractory mutation system, ARMS）方法或Sanger测序法检测。*EML4-ALK*融合基因通过Ventana免疫组织化学法（immunohistochemistry, IHC）（D5F3抗体）检测。本研究中患者的总生存期（overall survival, OS）是手术患者术后疾病复发时间或晚期患者的初诊时间至死亡时间或本研究结束时间计算的。

### 1.2 统计学处理

采用SPSS 19.0统计软件，计量资料应用*χ*^2^检验，对于理论频数 < 5的则采用*Fisher*确切概率法进行检验；计数资料采用两样本比较的非参数秩和检验（*Mann-Whitney U*检验），生存分析采用*Kaplan-Meier*法，并绘制生存曲线。采用*Cox*风险回归模型分析多个可能影响预后的因素；以*P* < 0.05判定有统计学差异。

## 2 结果

### 2.1 患者特征

本研究总计有553例NSCLC患者入组。中位年龄59岁，范围26岁-88岁；男性298例（53.9%），吸烟者239例（43.2%），腺癌患者525例（94.9%），Ⅳ期患者504例（91.1%）。

基因分型：553例NSCLC患者均进行了*EGFR*和*ALK*基因检测，*EGFR*突变率为41.0%（227/553），*ALK*重排的阳性率为10.5%（58/553），2例（0.4%）同时具有*EGFR*突变和*ALK*重排。患者的临床特征及基因分型情况见表 1（注：2例双突变患者未纳入下文中*EGFR*突变及ALK重排患者的数据分析）。

**1:** 553例患者的一般临床特征 Clinical characteristics of the study patients

Characteristic	Case (*n*=553)	Percentage (%)
Age (yr)		
Median	59	
Range	26-88	
< 65	372	67.3
≥65	181	32.7
Gender		
Male	298	53.9
Female	255	46.1
Smoking		
Yes	239	43.2
No	314	56.8
PS score		
0-1	522	94.4
≥2	31	5.6
Histology		
Adenocarcinoma	525	94.9
Squamous	22	4.0
NSCLC NOS	3	0.5
Mixed type	2	0.4
Large cell lung cancer	1	0.2
Stage		
Ⅲb	49	8.9
Ⅳ	504	91.1
Genetic typing		
*EGFR* mutation	227	41.0
ALK rearrangement	58	10.5
Co-mutation	2	0.4
Wild type	266	48.1
NSCLC: non-small cell lung cancer; NOS: not otherwise specified; EGFR: epidermal growth factor receptor; ALK: anaplastic lymphoma kinase; Co-mutation: *EGFR* mutation and *ALK* rearrangement; PS: performance status.		

### 2.2 不同基因分型患者的临床特征

227例*EGFR*突变患者中，男性87例（38.3%），女性140例（61.7%）；吸烟患者62例（27.3%），不吸烟患者165例（72.7%）；腺癌患者225例（99.1%），非腺癌患者2例（0.9%）。与*EGFR*无突变患者相比，227例*EGFR*突变患者在性别、吸烟史和病理类型方面均有统计学意义（*P*均 < 0.001），*EGFR*突变多见于女性、不吸烟、腺癌患者。58例ALK阳性患者中，男性30例（51.7%），女性28例（48.3%）；吸烟患者20例（34.5%），不吸烟患者38例（65.5%）；腺癌患者58例（100.0%）。ALK阳性与ALK阴性患者相比，年龄和病理类型方面差异均有统计学意义，更多见于年轻、腺癌患者（*P* < 0.001, *P*=0.002），而在性别、吸烟史、PS评分方面无显著差异（表 2）。

**2:** 不同基因突变患者的临床特征 Clinical characteristics of genotype specific subsets of patients

Characteristic	Genotype [*n* (%)]			*P*	
	EGFR (*n*=227)	ALK (*n*=58)		EGFR *vs* non-EGFR (*n*=227 *vs* *n*=324)	ALK *vs* non-ALK (*n*=58 *vs* *n*=493)
Age (yr)				0.400	< 0.001
Median	59	53			
< 65	157 (69.2)	52 (89.7)			
≥65	70 (30.8)	6 (10.3)			
Gender				< 0.001	0.677
Male	87 (38.3)	30 (51.7)			
Female	140 (61.7)	28 (48.3)			
Smoking status				< 0.001	0.125
Yes	62 (27.3)	20 (34.5)			
No	165 (72.7)	38 (65.5)			
PS score				0.108	0.770
0-1	218 (96.0)	54 (93.1)			
≥2	9 (4.0)	4 (6.9)			
Histology				< 0.001	0.002
Adenocarcinoma	225 (99.1)	58 (100.0)			
Non-adenocarcinoma	2 (0.9)	0 (0)			

### 2.3 所有患者的总生存

所有患者随访至2016年9月30日，共有345例（62.4%）患者死亡（包括111例*EGFR*突变患者、39例ALK阳性患者、1例双突变患者和194例野生型患者），有208例（37.6%）患者在研究结束时仍存活（包括116例*EGFR*突变患者、19例ALK阳性患者、1例双突变患者和72例野生型患者）。553例患者的中位生存期为17.2个月（95%CI: 14.290-20.110）。1例死亡的双突变患者未接受靶向治疗，仅接受含铂双药化疗4个周期，OS为6.2个月；另外1例双突变患者接受了化疗、靶向治疗，OS为21.0个月，目前仍存活。

553例患者生存进行单因素分析，包括年龄、性别、吸烟史、PS评分、病理类型、基因分型（551例患者）以及是否接受过靶向治疗等特征进行分析，结果表明女性、不吸烟、PS评分为0分-1分、腺癌、*EGFR*基因突变、接受过靶向治疗的患者较男性、吸烟、PS评分≥2分、非腺癌、EGFR野生型、未接受过靶向药物治疗的患者相比能显著延长生存期。551例患者*Cox*多因素分析显示PS评分（HR=2.640; 95%CI: 1.777-3.920; *P* < 0.001）、是否有*EGFR*突变（HR=1.607; 95%CI: 1.223-2.112; *P*=0.001）、是否靶向治疗（HR=1.584; 95%CI: 1.209-2.075; *P*=0.001）是影响复发或转移NSCLC患者OS的独立预后因素（表 3，图 1）。

**3:** 553例NSCLC患者生存单因素与多因素分析 Univariate and multivariate of the 553 patients with NSCLC

Characteristic	*n*	Events	OS (mo)	Univariate			Multivariate	
				95%CI	*P*		*P*	HR (95%CI)
Age (yr)					0.775			
< 65	372	236	18.0	15.085-20.915				
≥65	181	109	14.4	9.650-19.150				
Gender					< 0.001		0.185	0.822 (0.615-1.098)
Male	298	206	13.8	11.558-16.042				
Female	255	139	21.3	16.982-25.618				
Smoking status					0.002		0.911	0.984 (0.738-1.311)
Yes	239	158	14.6	12.252-16.948				
No	314	187	20.5	16.522-24.478				
PS score					< 0.001		< 0.001	2.640 (1.777-3.920)
0-1	522	317	18.7	15.562-21.838				
≥2	31	28	5.7	1.228-10.172				
Histology					0.023		0.668	1.116 (0.676-1.843)
Adenocarcinoma	525	328	17.8	14.990-20.610				
Non-adenocarcinoma	28	17	10.0	4.059-15.941				
*EGFR* mutation					< 0.001		0.001	1.607 (1.223-2.112)
Yes	227	111	28.7	22.160-35.240				
No	324	233	12.5	11.102-13.898				
*ALK* rearrangement					0.573			
Yes	58	39	15.5	10.991-20.009				
No	493	305	17.8	14.708-20.892				
TKI therapy					< 0.001		0.001	1.584 (1.209-2.075)
Yes	212	109	31.4	24.009-38.791				
No	341	236	12.7	11.425-13.975				

**1:**
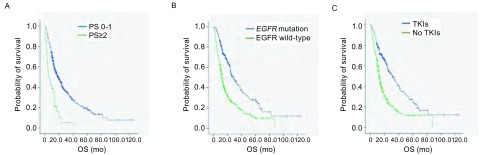
533例晚期NSCLC患者的*Kaplan-Meier*生存分析。A：PS为0分-1分和PS≥2分患者的中位OS比较（18.7个月*vs* 5.7个月，*P* < 0.001）；B：*EGFR*突变型患者和EGFR野生型患者的中位OS比较（28.7个月*vs* 12.5个月，*P* < 0.001）；C：接受靶向治疗的患者与未接受靶向治疗的患者的中位OS比较（31.4个月*vs* 12.7个月，*P* < 0.001）。 The *Kaplan-Meier* survival analysis of 533 patients with advanced NSCLC. A: Median OS in PS score (0-1) and PS score (≥2) patients (18.7 mo *vs* 5.7 mo, *P* < 0.001); B: Median OS in *EGFR* mutation and EGFR wild-type patients (28.7 mo *vs* 12.5 mo, *P* < 0.001); C: Median OS in treated with TKIs and untreated with TKIs patients (31.4 mo *vs* 12.7 mo, *P* < 0.001). TKIs: tyrosine kinase inhibitors; OS: overall survival.

*EGFR*突变患者的生存：227例*EGFR*突变的患者中，有111例（48.9%）患者死亡和116例（51.1%）患者在研究结束时仍存活，中位生存期为28.7个月（95%CI: 22.160-35.240）。单因素分析显示PS评分、是否EGFR-TKI靶向治疗是患者生存的影响因素，PS评分为0分-1分、接受靶向治疗的患者生存期明显延长（30.0个月*vs* 12.0个月，38.0个月*vs* 16.6个月），且有统计学差异（*P* < 0.001, *P* < 0.001）（图 2A）。*Cox*多因素分析显示PS评分为0分-1分（HR=4.451; 95%CI: 2.112-9.382; *P* < 0.001）、接受EGFR-TKI靶向治疗（HR=2.785; 95%CI: 1.871-4.145; *P* < 0.001）能显著延长患者的生存期，是影响*EGFR*突变患者生存的独立危险因素（表 4）。

**2:**
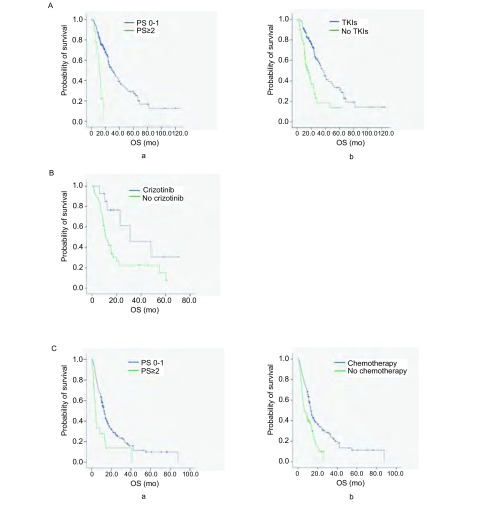
不同基因型晚期NSCLC患者的*Kaplan-Meier*生存分析。A：*EGFR*突变型患者的生存曲线，a：PS评分为0分-1分和PS评分≥2分患者的中位OS比较（30.0个月*vs* 12.0个月，*P* < 0.001）；b：接受靶向治疗的患者与未接受靶向治疗患者的中位OS比较（38.0个月*vs* 16.6个月, *P* < 0.001）；B：ALK阳性患者的生存曲线，接受克唑替尼治疗和未接受克唑替尼治疗患者的中位OS比较（31.0个月*vs* 11.0个月，*P*=0.022）；C：野生型患者的生存曲线，a：PS评分为0分-1分和PS评分≥2分患者的中位OS比较（12.6个月*vs* 3.5个月，*P* < 0.001）；b：接受化疗的患者与未接受化疗患者的中位OS比较（13.2个月*vs* 6.4个月，*P* < 0.001）。 The *Kaplan-Meier* survival analysis of patients with different genotype advanced non-small cell lung cancer. A: Survival curves of the *EGFR* mutation patients, a: Median OS in PS score (0-1) and PS score (≥2) patients (30.0 mo *vs* 12.0 mo, *P* < 0.001); b: Median OS in treated with TKIs and untreated with TKIs patients (38.0 mo *vs* 16.6 mo, *P* < 0.001); B: Survival curves of the ALK positive patients, median OS in treated with crizotinib and untreated with crizotinib patients (31.0 mo *vs* 11.0 mo, *P*=0.022); C: Survival curves of the wild-type patients. a: Median OS in PS score (0-1) and PS score (≥2) patients (12.6 mo *vs* 3.5 mo, *P* < 0.001); b: Median OS in treated with chemotherapy and untreated with chemotherapy patients (13.2 mo *vs* 6.4 mo, *P* < 0.001).

**4:** 227例*EGFR*突变的NSCLC患者生存的单因素与多因素分析 Univariate and multivariate of the 227 patients with NSCLC of *EGFR* mutation

Characteristic	*n*	Events	OS (mo)	Univariate			Multivariate	
				95%CI	*P*		*P*	HR (95%CI)
Age (yr)					0.472			
< 65	157	82	26.5	19.038-33.962				
≥65	70	29	31.4	16.192-46.608				
Gender					0.081			
Male	87	47	25.0	22.127-27.873				
Female	140	64	36.0	22.452-49.548				
Smoking status					0.995			
Yes	62	27	28.7	22.454-34.946				
No	165	84	29.0	18.446-39.554				
PS score					< 0.001		< 0.001	4.451 (2.112-9.382)
0-1	218	103	30.0	23.143-36.857				
≥2	9	8	12.0	6.741-17.259				
EGFR-TKI therapy					< 0.001		< 0.001	2.785 (1.871-4.145)
Yes	158	69	38.0	30.781-45.219				
No	69	42	16.6	11.285-21.915				
Line of TKI therapy					0.542			
First line	102	34	38.0	25.996-50.004				
Others line	56	35	36.0	26.349-45.651				

ALK阳性患者的生存：58例ALK阳性患者中，41例（67.2%）死亡，中位生存期为15.5个月（95%CI: 10.991-20.009）。单因素分析表明ALK阳性患者的生存期仅与是否接受克唑替尼治疗有关，接受克唑替尼治疗的患者中位OS为31.0个月，未接受克唑替尼治疗的患者中位OS为11.0个月，两者差异有统计学意义（*P*=0.022）（图 2B），而在年龄、性别、吸烟史、PS评分以及克唑替尼治疗线数方面生存期均无统计学差异（表 5）。

**5:** 58例ALK阳性NSCLC患者的单因素分析 Univariateof the 58 patients with NSCLC of ALK positive

Characteristic	*n*	Events	OS (mo)	Univariate	
				95%CI	P
Age (yr)					0.237
< 65	52	37	13.6	8.640-18.560	
≥65	6	2			
Gender					0.961
Male	30	20	15.5	10.812-20.188	
Female	28	19	12.3	4.530-20.070	
Smoking status					0.397
Yes	20	14	13.0	7.153-18.847	
No	38	25	15.5	7.890-23.110	
PS score					0.184
0-1	54	35	15.5	11.188-19.812	
≥2	4	4	1.9	0.000-20.422	
Crizotinib treatment					0.022
Yes	14	6	31.0	1.829-60.171	
No	44	33	11.0	7.354-14.646	
Line of crizotinib					0.715
First line	5	2	31.0		
Others line	9	4	48.0	0-101.428	

野生型患者的生存：在266例野生型患者中，有194例（72.9%）患者死亡和72例（27.1%）患者在研究结束时仍存活。中位生存期是12.1个月（95%CI: 10.660-13.540）。单因素分析显示只有PS评分和治疗方式对患者的生存有影响，PS评分为0分-1分、接受化疗的患者生存期明显延长（12.6个月*vs* 3.5个月，13.2个月*vs* 6.4个月），且有统计学差异（*P* < 0.001, *P* < 0.001）（图 2C）。*Cox*多因素分析显示PS评分为0分-1分（HR=2.313; 95%CI: 1.380-3.877; *P*=0.001）、接受化疗（HR=1.911; 95%CI: 1.396-2.616; *P* < 0.001）是影响野生型患者OS的独立危险因素（表 6）。

**6:** 266例野生型NSCLC患者生存的单因素与多因素分析 Univariate and multivariate of the 266 patients with NSCLC of wild type

Characteristic	*n*	Events	OS (mo)	Univariate			Multivariate	
				95%CI	*P*		*P*	HR (95%CI)
Age (yr)					0.587			
< 65	161	116	13.0	10.880-15.120				
≥65	105	78	11.7	9.854-13.546				
Gender					0.125			
Male	179	138	11.9	10.076-13.724				
Female	87	56	14.0	11.229-16.771				
Smoking status					0.254			
Yes	155	116	12.0	10.238-13.762				
No	111	78	13.0	10.266-15.734				
PS score					< 0.001		0.001	2.313 (1.380-3.877)
0-1	248	178	12.6	10.984-14.216				
≥2	18	16	3.5	2.045-4.955				
Histological type					0.536			
Adenocarcinoma	240	178	12.3	10.910-13.690				
Non-adenocarcinoma	26	16	9.0	2.023-15.977				
Chemotherapy					< 0.001		< 0.001	1.911 (1.396-2.616)
Yes	188	133	13.2	11.602-14.798				
No	78	61	6.4	2.722-10.078				
Targeted treatment					0.400			
Yes	39	34	9.0	5.533-12.467				
No	227	160	12.7	11.102-14.298				

基因突变患者与野生型患者的生存比较：有基因突变且接受靶向治疗的患者共172例（158例*EGFR*突变患者和14例ALK阳性患者），中位OS为36.0个月，与野生型患者相比，生存期差异有统计学意义（*P* < 0.001）。有基因突变未接受靶向治疗的患者共113例（69例*EGFR*突变患者和44例ALK阳性患者），中位OS为13.6个月，与野生型患者相比，生存期差异无统计学意义（*P*=0.181）（图 3）。

**3:**
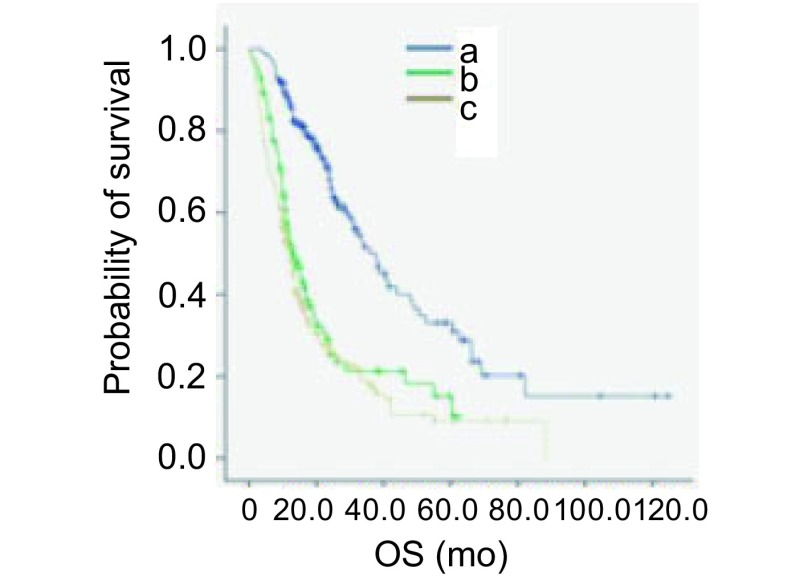
基因突变患者是否接受靶向治疗与野生型患者的生存曲线比较。有基因突变且接受靶向治疗的患者和野生型患者的中位OS比较（36.0个月*vs* 12.1个月，*P* < 0.001），有基因突变未接受靶向治疗的患者和野生型患者的中位OS比较（13.6个月*vs* 12.1个月，*P*=0.181）。 Comparison of survival curves between patients harboring gene mutation treated with TKI or not and wild-type patients. Median OS inpatients harboring gene mutation treated with TKI and wild-type patients (36.0 mo *vs* 12.1 mo, *P* < 0.001). Median OS in patients harboring gene mutation untreated with TKI and wild-type patients (13.6 mo *vs* 12.1 mo, *P*=0.181).

## 3 讨论

近些年来，多项研究表明针对驱动基因的靶向治疗在晚期NSCLC患者的治疗和预后方面产生了革命性的影响。*EGFR*和*ALK*是NSCLC最重要的两个驱动基因。研究^[14-17]^表明，*EGFR*基因突变在亚裔、女性、非吸烟、腺癌患者中发生率较高，*EGFR*突变在中国肺腺癌患者中的突变率为51%。在大样本的晚期NSCLC患者中同时分析*EGFR*突变和*ALK*重排患者及野生型患者生存的研究较少。本研究中553例晚期NSCLC患者有227例患者发生*EGFR*突变（不包括2例双突变患者），突变率为41.0%，且与EGFR野生型相比，*EGFR*突变多见于女性、不吸烟、腺癌患者中（*P*均 < 0.001），与以前的研究结果^[18]^基本一致。在NSCLC中ALK染色体易位的发病率已经在3%-13%的西方国家和中国人中被报道^[19-22]^。*ALK*融合基因在年轻、不吸烟或很少吸烟、腺癌患者中更常见^[22]^。本研究中*ALK*重排的阳性率为10.5%（58/553），这与已经发表过的文章中的结果相似^[19, 23]^，而且与ALK阴性患者相比更多见于年轻、腺癌患者（*P* < 0.001, *P*=0.002），与其他学者的研究^[19, 22, 24]^报道一致。

在总人群的生存分析中，单变量分析表明女性、不吸烟、PS评分为0分-1分、腺癌、*EGFR*基因突变、接受过靶向治疗是较好生存结果的预测因素。*Cox*多因素回归分析显示PS评分、是否有*EGFR*突变、是否靶向治疗是影响晚期NSCLC患者总生存期的独立预后因素，而与性别、吸烟史、病理类型、*ALK*基因是否发生融合等因素无关。多项研究^[25, 26]^显示，*EML4-ALK*融合基因突变组与未突变组患者比较，OS差别无统计学意义，与本研究结果一致。

近年来大量临床研究^[4-8]^证明*EGFR*基因突变患者接受EGFR-TKI治疗的临床疗效明显优于化疗，同传统化疗相比能明显改善患者的PFS，但并未延长OS，与试验两组交叉接受化疗与靶向治疗有关，但Lux-lung6^[8]^显示阿法替尼能显著延长19突变患者的OS。本研究中227例*EGFR*突变患者的中位OS为28.7个月（95%CI: 22.160-35.240）。*Cox*分析表明PS评分、是否接受EGFR-TKI靶向治疗是*EGFR*突变患者生存的独立危险因素，而与年龄、性别、吸烟史、病理类型这些因素均无关。

针对*EML4-ALK*融合基因开发的靶向药物克唑替尼在2011年获得美国美国药品食品管理局（Food and Drug Administration, FDA）批准上市，用于治疗ALK阳性的晚期NSCLC患者。在PROFILE 1014 Ⅲ期临床试验中比较克唑替尼和含铂双药标准化疗一线治疗ALK阳性NSCLC的疗效，结果表明克唑替尼比化疗能明显延长患者的PFS（10.9个月*vs* 7.0个月）^[12]^。本研究中58例ALK阳性患者的中位OS是15.5个月（95%CI: 10.991-20.009），分析表明ALK阳性患者的生存期仅与是否接受克唑替尼治疗有关。14例接受克唑替尼治疗患者的中位OS是31.0个月（95%CI: 1.829-60.171），这与Show等^[27]^的Ⅰ期临床试验中接受克唑替尼治疗的患者较未接受克唑替尼治疗患者的生存期明显延长的结果是相似的。所以，本研究中ALK阳性患者中位OS显著低于*EGFR*突变患者，考虑与仅有24%（14/58）的患者服用克唑替尼有关。虽然多项临床试验^[28-30]^表明克唑替尼能延长ALK阳性患者的PFS，但目前尚无研究表明是否克唑替尼靶向治疗是ALK阳性患者的独立危险因素，同样与试验中治疗高度交叉有关。

本研究中266例野生型患者的预后与PS评分及是否接受化疗有关，其中接受化疗患者的OS显著优于未接受化疗患者。目前，化疗联合或不联合抗血管药物是野生型肺癌患者的主要治疗方法，随着靶向药物研发的进展，对于EGFR和ALK野生型患者，可进行*ROS1*、*c-MET*、*BRAF*等基因检测，有上述突变患者也可选择相应的靶向药物治疗，有助于改善患者的预后。新兴的免疫检查点抑制剂在野生型肺癌患者也有很好的疗效^[31]^。

综上所述，不同基因分型的晚期NSCLC的预后有较大差异，临床上应根据基因分型结果，并结合患者的年龄、体能状况、病理类型等情况制定个性化治疗方案，从而更有利于控制疾病的发展及延长患者的OS。
